# The efficacy of dexamethasone on reduction in the reoperation rate of chronic subdural hematoma – the DRESH study: straightforward study protocol for a randomized controlled trial

**DOI:** 10.1186/1745-6215-15-6

**Published:** 2014-01-06

**Authors:** Stephan Emich, Bernd Richling, Marc R McCoy, Rahman Abdul Al-Schameri, Feng Ling, Liyong Sun, Yabing Wang, Wolfgang Hitzl

**Affiliations:** 1 Christian Doppler Klinik, PMU Salzburg, Universitätsklinik für Neurochirurgie, Ignaz Harrer Str. 79, 5020 Salzburg, Austria; 2 PMU Salzburg, Strubergasse 21, 5020 Salzburg, Austria; 3 Christian Doppler Klinik, PMU Salzburg, Division Neuroradiologie, Universitätsklinik für Radiologie, Ignaz Harrer Str. 79, 5020 Salzburg, Austria; 4 Department of Neurosurgery, Xuanwu Hospital, Capital Medical University Beijing, 45 Changchun Street, 100053 Beijing, PR China

**Keywords:** Chronic, Subdural hematoma, Glucocorticoid, Dexamethasone, Therapy, Operative, Treatment

## Abstract

**Background:**

Chronic subdural hematoma (cSDH) is a common neurosurgical disease. It is often considered to be a rather benign entity. In spite of well established surgical procedures cSDH is complicated by a recurrence rate up to 30%. Since glucocorticoids have been used for treatment of cSDH in 1962 their role is still discussed controversially in lack of evident data. On the basis of the ascertained inflammation cycle in cSDH dexamethasone will be an ideal substance for a short lasting, concomitant treatment protocol.

Objective: to test the efficacy of dexamethasone on reduction inthe reoperation rate of cSDH.

**Methods/Design:**

The study is designed as a double-blind randomized placebo-controlled trial 820 patients who are operated for cSDH and from the age of 25 years are included after obtaining informed consent. They are randomized for administration of dexamethasone (16-16-12-12-8-4 mg/d) or placebo (maltodextrin) during the first 48 hours after surgery. The type I error is 5% and the type II error is 20%. The primary endpoint is the reoperation within 12 weeks postoperative.

**Discussion:**

This study tests whether dexamethasone administered over 6 days is a safe and potent agent in relapse prevention for evacuated cSDH.

**Trial registration:**

EudraCT 201100354442

## Background

Chronic subdural hematoma (cSDH) is a common neurosurgical disease: Its one-year incidence rate is about 5 per 100,000 in the general population, but increases for those aged 70 years and older (58 per 100,000 per year) [1]. Because the proportion of people aged 65 years and older is expected to double worldwide between 2000 and 2030 [2], a large rise in incidence is expected.

Despite its relatively high mortality and morbidity it is often considered to be a rather benign entity [3,4]. The main risk defining the outcome is recurrence. Even if operated by skillful neurosurgeons, recurrence rates from 3.8 to 30% are reported [4-6].

cSDH was first described by JJ Wepfer [7] in 1656, followed by Rudolf Virchow, [8] who detected an inflammatory element and in 1856 named this condition *pachymeningitis haemorrhagica interna*. The formation of new membranes and the extravasation of fluid in the cavity between these membranes and layers was seen by Virchow as typical for this disease. Today’s definition of chronic subdural hematoma as a 'chronic, self-perpetuating inflammatory process that involves the dura mater' was given by Frati [9] and confirms the inflammatory nature of this disease.

Several causes have been described as mechanisms of origin. Analogous to acute subdural hematoma caused by trauma, the occurrence of chronic subdural hematoma has often been described in conjunction with head injury. Histopathological evidence for the influence of trauma was given by Schachenmayr [10] in 1978 describing that after microtrauma a cleavage of the inner dural layer occurs ('tissue torn artifact'). Still today the role of head trauma in the origin of chronic subdural hematoma is not clear. A literature search discloses that at least mild head trauma had occurred in 8% [11] to 80% [12] of the patients.

An important histopathological mechanism of origin is the high non-specific reaction potential of the capillary network in the inner dural sheet which, coming in contact with blood or fibrin degredation products, leads to the formation of a neomembrane. This mechanism is supported by the inflow of liquids like blood, later plasma and/or cerebrospinal fluid (CSF. Further progression is maintained by tissue activator (TA) exuding from the extremely vascularized membranes into the cavity, perpetuating a fibrin/fibrinogene mechanism leading to continuous microhemorrhages. Furthermore the automembrane developes inflammatory cells, like mastcells, eosinophiles, neutrophiles, monocyts, macrophages, endothelial cells and fibroblasts, being continuously activated and recruited. This constitutes a source of inflammatory angiogenic fibrinolytic and coagulation factors [9].

The symptomatology consists of headache, neurological focal symptoms, aphasia, epilepsy and unconsciousness. After assessment of the symptoms a neurological classification can be made grading the patients from grade 0 to grade 4 [12]. The main diagnosis however, is done by imaging. Computerized tomography (CT) and magnetic resonance imaging (MRI) disclose monolateral or bilateral extracerebral fluid with more or less membranes separating the hematoma into chambers. Different classifications for both imaging tools are available [13-15].

The primary therapeutic option is surgery. The standard procedure consists of a burr hole above the site of the lesion with or without irrigation. Santarius [16] has proved that an insertion of a drain diminishes recurrence and mortality at 6 months after burr hole craniostomy. In cases of separation of the hematoma by thick layers, open craniotomy must be chosen to allow for evacuation of all chambers [17]. Recurrence of the hematoma is defined as reappearance of the clinical and radiological symptoms and leads to reoperation.

The first remark about a nonsurgical treatment of cSDH was given by Ambrosetto [18] in 1962. He described four patients, who received a conservative multimodal therapy in combination with corticosteroids: Their clinical symptoms regressed completely. Frati [9] and Labadie [19,20] reported that due to the proof of proinflammatory and inflammatory cytogene that anti-inflammatory therapy may be beneficial. However, several national surveys show a controversial use of cortisone in treatment of cSDH: 38% of French neurosurgeons use adjuvant corticosteroid therapy after surgery [21]. For 13% of Canadian neurosurgeons cortisone plays a role in the treatment of cSDH [22], and 55% of surgeons in the UK and Ireland prescribe steroids in conservatively managed patients [23]. Berghauser Pont [24] wrote about a generous use of corticosteroids in a nonsurgical setting and in preparation for surgery respectively, in combination with surgical treatment in the Netherlands.

There are only poor data published about the efficacy of concomitant cortisone medication. Sun [25] describes 112 cases treated in a prospective set-up. A systematic review regarding corticosteroids and cSDH [26] found that beyond Sun’s trial there are only four retrospective studies and concludes that steroids are beneficial and suggests more prospective data are needed. Berghauser Pont also performed a retrospective cohort study [27] of patients with cSDH on preoperative corticosteroid therapy. If there were no signs of recovery patients underwent surgery: 496 patients with burr hole craniostomy were enrolled. Recurrence was stated at 11.9. In spite of preselection the basis of cortisone therapy, the relapse rate is low.

In 2011 Park published a prospective study about cSDH [28]: 31 patients underwent burr hole irrigation plus external drainage. The objective of this study was to investigate the relationship between fibrinolytic factors and computed tomography findings. We emphasize in this trial the postoperative administration of dexamethasone for one week, because the reoperation rate Park described was only 3.2.

Thus, the DRESH study will be the first randomized controlled trial (RCT) to evaluate the role of a short postoperative cortisone protocol in the operative treatment of cSDH.

### Choice of comparator

Dexamethasone has been selected because contrary to methylprednisolone, it is independent of weight, allowing for a simplified randomization and blinding procedure. Other advantages of dexamethasone are the longest biological half-life of all glucocorticoids (36 to 54 hours) and seven times higher potency than prednisolone. The total dosage used in the DRESH study is 68 mg in Austria and 68.25 mg in China, which is equivalent to a two times higher dosage of prednisolone (1 mg/kg/day). The drug concentration in CSF correlates with the peak concentration in plasma. The DRESH study considers these pharmacokinetics by a single dose at a physiological time in the morning.

### Study objectives

#### Primary objective

The primary objective is to demonstrate that dexamethasone reduces the reoperation rate by 50% within 12 weeks after hematoma evacuation.

#### Secondary objectives

The secondary objectives of the study are as follows: to measure the effect of dexamethasone on clinical outcome by the Markwalder score during a period of 12 weeks after surgery; the impact on radiologic signs of the hematoma, such as thickness, membranes and mass-demanding effect (midline-shift); to evaluate risk factors of the genesis and recurrence of cSDH in a prospective study design; risk of infectious complications, for example, pneumonia, urinary tract infection, ventriculitis, meningitis and encephalitis; dexamethasone-dependent severe psychiatric reactions; and death.

### Trial design

This is a phase III, prospective, multi-center, double blind, randomized, placebo-controlled study. Eligible participants are randomized in a 1:1 allocation ratio to one of the two arms: an intervention arm, in which the participants receive dexamethasone according to the protocol, and a control arm in which they receive placebo.

## Methods

### Study setting and participants

Patients will be recruited from 19 Departments of Neurosurgery in China that are in close contact to the Capital Medical University Beijing (Table 1) and from the Department of Neurosurgery, Paracelsus Medical University Salzburg, Austria. The study is the first common project of cooperation between both medical universities.

**Table 1:** List of Chinese study cites

	**Address of hospital**	**ZIP code**
1	Jiangsu Province Xuzhou Hospital	221009
2	Neimeng Medical University 1st Affiliated Hospital	22150
3	Shanxi Province Datong 5th Hospital	37006
4	Gansu Province Wuwei Hospital	733000
5	Neimeng Province Linye Hospital	22150
6	Shanxi Province Liangzhou Hospital	726000
7	Guangdong Province Baoan Central Hospital	518101
8	Hebei Province Tangshan Central Hospital	63300
9	Heibei Province Tang Country 1st Hospital	262400
10	Qing Hai Province People Hospital	810007
11	Guizhou Province Zunyi Central Hospital	441000
12	Henan Province Anyang Local Hospital	455000
13	Guangxi Province 1st Hospital	455000
14	Guangdong Province Huizhou 1st Hospital	530021
15	Shandong Province Qingdao 1st Hospital	516000
16	Shandong Province Longkou Central Hospital	266400
17	Shandong Province Cangshan People’s Hospital	265701
18	PLA 2nd Hospital	100037
19	Harbin Medical University 4th Affiliated Hospital	431700

### Inclusion criteria

The inclusion criteria are: 1) patients who are to undergo surgery for cSDH according to one or both of the following criteria: clinical symptomatology (Markwalder grade 1 to 4) with correlation to the cerebral lesion, or radiologic finding of cSDH with flattening of the underlying cortex and/or midline shift more than 5 mm; and 2) male or female patients aged over 25 years (inclusive).

### Exclusion criteria

The exclusion criteria are: 1) pregnancy and nursing; 2) diabetes; 3) hypertension (systolic blood pressure (SBP) >180 mmHg) that is refractory to treatment; 4) glaucoma; 5) Known hypersensitivity to dexamethasone; 6) known peptic ulceration; 7) acute systemic infection (fever, leucocytosis, elevated C-reactive protein (CRP) and/or PCT (Procalcitonin); 8) parasitic infection; 9) current or previous history of severe affective disorders (especially previous steroid psychosis); 10) recent myocardial infarction (as myocardial rupture has been reported); 11) patients for whom study drug cannot be started within 48 hours after evacuation of the hematoma; 12) patients for whom it is known at the time of screening that certain follow-up, protocol-mandated imaging assessments will not be feasible; 13) patients unlikely to comply with the protocol (for example, unable to return for follow-up visits); 14) current alcohol or drug abuse, or dependence; 15) use of another investigational product within 28 days prior to randomization; 16) vitamin-K antagonists during the first 2 weeks after operation; 17) GpIIb/IIIa-receptor antagonists, ASS (acetylsalicylic acid) or NOAC (new oral anticoagulants) during the first two weeks after surgery; and 18)reoperation within 48 hours after the first operation.

### Interventions

#### Investigational drug

The intervention which will be evaluated in this trial is the intake of dexamethasone. In Austria the tablets are provided in capsules of 4 mg each and are taken orally before 8.00 in the morning. The daily dosages are 16 mg at days 1 and 2, 12 mg at days 3 and 4, 8 mg at day 5, and 4 mg at day 6. In China there are only tablets of 0.75 mg dexamethasone available, so capsules are filled with six, four or three tablets. The following dosages can be obtained: 15.75 mg at days 1 and 2; 12 mg at days 3 and 4; 8.25 mg at day 5 and 4.5 mg at day 6. This intervention will be compared against placebo.

#### Comparative drug

Matching oral capsules are filled with maltodextrin to the same weight and are administered similarly.

#### Concomitant care

All patients in the study will receive the routine standard of care. This will include a check of blood and vital parameters, a neurologic investigation and CT scan at admission. An acute onset of neurologic deterioration or a decline of alertness will need an urgent operative treatment. For neurosurgical procedures normal coagulation parameters are essential. In warfarin medication, the international normalized ratio (INR) has to be normal prior to surgery. Coagulation factors or other blood products will be administered as required. ASS is to be stopped five days and GpIIb/IIIa-receptor antagonists seven days before surgery, and NOAC a duration longer than double of their pharmacological half-life. Only those patients are eligible who are able either to pause their anticoagulant or antiplatelet medication, or to tolerate a conversion to LMWH (low molecular weight heparin) for two weeks postoperatively.

In consideration of different techniques available for the evacuation of a chronic subdural hematoma, the following procedure has been agreed: one burr hole over the site of the hematoma, no irrigation and a closed drainage system. The drain will be continued up to 48 hours. Criteria for discontinuing before 48 hours are air in the system, an incorrigible flow arrest or delivery of pure CSF.

Pain will be treated according to the WHO guidelines [29].

LWMH are used for deep venous thrombosis prophylaxis. The application has to start within 24 hours postoperatively, or if the patient is mobile, less than 3 hours a day. Daily neurologic investigations and control of vital parameters and wound healing will be added to standard care, as will concerted medication and drawing of blood samples, CT scans and physical therapy as necessary.

There will be a focus on hyponatremia and pathologic INR values. Patients who received oral anticoagulation preoperatively will need to be controlled daily and will have to have their parameters kept within the normal range throughout the hospital stay. The same procedure will be applied in hyponatremia. At study day-3 blood glucose levels are evaluated. At a level higher than 300 mg/dl the patient will be excluded from the trial. In order to detect a raised intraocular pressure a measurement will be performed during the treatment period.

Participants will remain inpatients until the study drug is administered completely. Patients who ready to be discharged home earlier will have to finish the medication on their own. Protocol-mandated assessments will be performed according to the table of assessments (Table 2). CT and/or MRI scans will be sent to a core laboratory and evaluated centrally in a triple-blinded way. Blood samples will be analyzed by the investigational laboratory locally.

**Table 2:** Visit and assessment schedule

**VISITS**	**Screening**	**Post-procedure**	**Day 2 post operation**	**Day 3 of drug protocol**	**Until hospital discharge**	**Week 4 (16±7 days)**	**Week 12 (48±7 days)**	**Week 24**^**1**^**(96±7 days)**
Informed consent	x							
Medical history/demographics	x							
Procedure related information	x							
Randomization	x							
Concomitant medications^2^	x	x	x	x	x	x		
Physical examination	x				x			
Hematology, biochemistry, coagulation^3^	x				x(OD)^4^			
Urine pregnancy test	x							
Markwalder score	x				x (OD)	x	x	x
Blood glucose				x				
Intraocular pressure					x			
Neurological worsening events					x	x	x	
CT scan	x^5^		x^6^			x	x	x
Post-discharge information						x	x^7^	x
Adverse events			x	x	x^8^			
Serious adverse events	x^9^	x^9^	x	x	x	x	x^10^	

OD=once a day.

CT=computer tomography.

^1^Only in case of reoperation.

^2^Concomitant medications will be recorded in the CRF from 2 weeks prior to study drug start and up to the end of study drug medication.

^3^Blood will be analyzed locally and the results will be recorded in the CRF.

^4^Preoperative oral anticoagulated patients need a coagulation monitoring once a day to keep the parameters normal. In the same way are patients monitored with hyponatremia.

^5^If the baseline CT scan performed at a referral center, is of acceptable quality, and is available at the investigational site at the time of screening, it does not need to be repeated. However, if the only baseline CT scan available is a hardcopy film, then it must be repeated at the investigational site.

^6^CT scan to be performed during 48h post operation. In case of neurological worsening CT scans are repeated as required.

^7^At week 12 the patient will return to the hospital for documentation of his/her residence (e.g. home, rehabilitation centre etc.), any new hospitalizations or surgical procedures (directly or indirectly related to the cSDH) since the first hospital discharge, and to ensure that ongoing AEs/SAEs or new SAEs are followed up/documented. In case of reoperation the same procedure is performed at week 24.

^8^AEs reporting and follow-up: Reporting of all new AEs up to one day after study drug discontinuation; follow-up of ongoing AEs only thereafter.

^9^SAEs related to study-mandated procedures after signature of informed consent and before study drug administration.

^10^Serious adverse events still ongoing at the Study Completion visit must be followed until resolution or stabilization or until the event is otherwise explained. New SAEs occurring at any time after the 21-day follow-up period after study drug discontinuation may be reported to the Sponsor within 24 hours of the investigator´s knowledge of the event, if felt appropriate by the investigators.

Two regular follow-up visits are planned. At weeks 4 and 12 after the operation a CT scan and clinical investigation are performed. Only in the case of reoperation is another visit necessary as an outpatient at week 24.

Concomitant medications will be proton pump inhibitors, vitamin K and anticonvulsants as required. In addition participants will have to avoid traditional medicines (that is, plant-, animal- or mineral-based medicine, such as traditional Chinese medicine).

### Outcomes

#### Primary Outcome Measures

It is considered that the prevention of reoperation would be the most important clinically primary endpoint.

#### Secondary Outcome Measures

Secondary outcomes include clinical evaluation by the Markwalder score and radiologic characteristics of the CT/MR images over time. On axial CT scans the thickness of the hematoma and midline shift is measured in millimeters at the thickest area and the volume is calculated in cubic centimeters. Midline shift is measured at the level of the intraventricular foramen. Hematomas are classified as homogenous, laminar, separated or trabecular. Their location is classified as being over the convexity, at the cranial base or the interhemispheric space. There is also a focus on brain atrophy that is classified as none, mild, definite, or severe. Residual air is measured in cubic centimeters. The influence of a patient’s long-term medication is evaluated together with coexisting diseases as predictors for recurrence.

### Participant timeline

**Figure 1:**
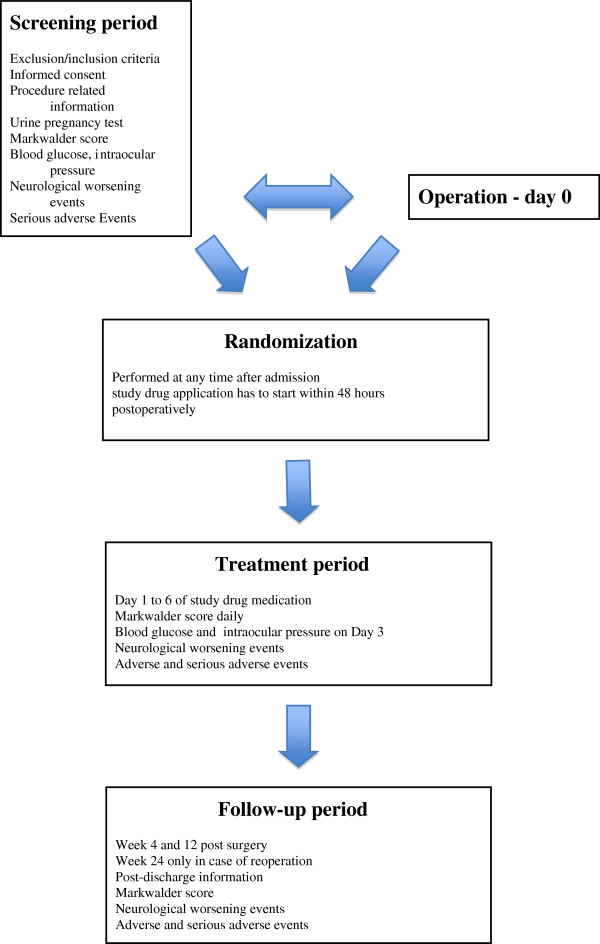
Flow diagram.

A time schedule of enrolment, interventions, assessments and visits for participiants are shown in Table 2. The flow diagram (Figure 1) illustrates the key steps of the trial.

### Sample size

Let p_verum_ be the reoperation rate in the verum group and p_placebo_ be the reoperation rate in the placebo group. The null hypothesis H_0_ is that the ratio of both rates p_placebo_ /p_verum_ is equal or smaller than 2, the corresponding alternative hypothesis H_1_ is that the ratio p_placebo_ /p_verum_ is larger than 2. In other words, the alternative hypothesis states that dexamethasone reduces the reoperation rate by 50% or more. For a reasonable medication the effect size is assumed to be this size because dexamethasone will be taken by the majority without benefit.

The assumed reoperation rate p_placebo_ is 10%. This is in accordance with the data from our department and the Chinese study sites. In order to detect a ratio p_placebo_ /p_verum_ of 2 or more with a power of 80%, the sample sizes are n1 = n2 = 370 in each arm. The type I error is 5% and the type II error for this scenario is 20%. The actual power of 83% is based on the exact power for the ratio of two binomial populations, one-sided and based on the score test (StatXact Cytel Software Corporation (2002) *StatXact 6.0 For Windows User Manual*, Cytel Software Corporation, Cambridge MA, USA). Due to possible drop-out of patients, we increased the sample sizes up to 410 in each group. Thus, 820 patients undergoing surgery for cSDH will be included.

### Randomization

Stratified randomization is done by using a computer randomization algorithm to generate balanced random samples (PASS 11, Hintze, J (2012). NCSS 8, NCSS, LLC. Kaysville, Utah, USA. http://www.ncss.com and Rosenberger, WF, and Lachin, JM 2002. *Randomization in Clinical Trials - Theory and Practice*. John Wiley & Sons. New York.) within each study site. Study site is the stratification variable for this randomization plan. Hence, the number of patients who receive verum medication is the same as the number of patients who receive a placebo in each study site, a 1:1 allocation ratio. Each patient receives a unique integer number ranging from 1 to 820 as an identification code, and this number will also be assigned to the corresponding medication kit. Each study site has a predefined set of patients to complete. In order to avoid consecutive recall of medication kits, the sequence in each study site is reordered by using random permutations.

The randomization plan will be implemented into an interactive web response system (IWRS) and hence the randomization is available for each study site. The investigators (or study staff) will interact with the system to receive the information as to which study group the patient is allocated, and which medication kit has to be used.

### Data analysis

The primary endpoint is the rate of reoperation between 48 hours and 12 weeks after the initial evacuation of a chronic subdural hematoma. Mandatory reoperation during the first 48 hours is related to surgical complications. In such rare cases the patient will be excluded.

Evaluation of the Markwalder score will be done by computing crosstabulation tables and applying Pearson’s chi-square test. Data will be checked carefully for outliers and for normality based on the Kolmogrov-Smirnov test and Liliefors test. To evaluate the impact of dexamethasone on the thickness of the hematoma and other continuously distributed, metrically scaled variables, mixed models with treatment group as the independent factor and time as the repeated factor will be applied (if all assumptions for the model are met). Linearity will be assessed by Box Tidwell and log transformation. Corresponding planned contrasts together with 95% confidence intervals will be computed. If these variables do not fulfill the assumptions for parametric testing, nonparametric methods such as the bootstrap *t*-test, Kruskal-Wallis analysis of variance (ANOVA) with median tests and Friedman ANOVA with Wilcoxon matched-pairs test will be applied: 95% confidence intervals based on Pearson-Clopper values will be computed to estimate the reoperation rate in both groups. An unconditional test (Barnard’s test for superiority) for the ratio of these two proportions will be used. A *P*-value less than 5% will be considered statistically significant. All analyses will be done by one of the authors (WH) using STATISTICA 10 (Hill, T and Lewicki, P (2011). *STATISTICS: Methods and Applications*. StatSoft, Tulsa, OK, USA), StatXact 6.0 (Cytel Software Corporation (2003) Cytel Software Corporation, Cambridge MA, USA), NCSS 8 and PASS 11 Hintze, J (2012), NCSS 8, NCSS, LLC, Kaysville, UT, USA. (http://www.ncss.com).

### Monitoring

#### Data monitoring

Standard operating procedures for key processes in the study have been developed. Prior to recruitment, the field team will receive extensive training on the objectives, methods and processes of the study, as well as on the concept of ethical research. All case report form (CRF) data for each participant will be controlled and signed by a second investigator at this study site. Subsequently, the entered data are systematically checked by medically trained personnel. Errors with obvious corrections will be corrected by the medically trained personnel and communicated to the site. The CRF will be inspected by a monitor at regular intervals throughout the study to verify the adherence to the protocol and the completeness, consistency and accuracy of the data being entered on them.

#### DSMB

A DSMB (Data Safety Monitoring Board) will ensure the monitoring of adverse events (AEs)/severe adverse events (SAEs) and adverse drug reactions (ADRs) during the study.

#### Waived SAEs

The decision to have waived SAEs is appropriate when the safety and tolerability profile of the study drug dexamethasone has been well-characterized, allowing a clear understanding of the difference between drug-related and disease-related AEs. The SAEs listed below are due to the underlying disease, and are therefore expected to occur in this patient population. In this study, they will be waived. This means that they will not require reporting to the sponsor drug safety database on an SAE form, but that they will be reported as serious only on the AE pages of the CRF. Therefore, they will be entered only into the Sponsor’s clinical database. The waived SAEs for this study are the following: 1) any type of cerebral/brain infarction or stroke; 2) cerebral/brain hemorrhage or hematoma; 3) delayed ischemic neurological deficit, neurological deterioration, delirium, confusion, disorientation, aphasia, paresis/paralysis (including hemiparesis, limb weakness, specified limb paralysis); 4) cranial nerve palsies; 5) complications related to the initial haematoma evacuation procedure; 6) brain edema; 7) hydrocephalus; 8) intracranial hypertension; 9) meningitis, ventriculitis, encephalitis; and 10) seizure.

#### Interim analysis

There will be no interim analysis performed.

#### Auditing

Quality control audits of the database will be made before study closure.

### Informed consent

All eligible patients will be presented with a form for written informed consent. The form will also be verbally explained. A signature will be obtained from all those who consent to participate respectively or from their legal representative. The written informed consent form will be in Chinese, English and German.

#### Ethical issues

The protocol received Salzburg Ethics Committee approval, and was approved by the BASG (Austrian health products safety agency). The trial is conducted in compliance with the European Union Clinical Trials Directive (2001/20/EC), the Public Health Code of Ethics, the International Conference on Harmonization guidelines for Good Clinical Practice (CPMP/ICH/135/95) and the principles of the Declaration of Helsinki (1996).

## Discussion of underlying literature

Despite 2,335 papers about cSDH identified in a recent PubMed search, treatment has not changed much. The main problem is the persistent recurrence. Glucocorticoids have a special capacity to block the inflammatory mechanism in the formation of the cSDH. They specifically inhibit lymphokins and prostaglandins and stimulate inflammatory inhibitors like lipocortin. Thus, the growing of neomembranes and neocapillaries is impaired [3,19,20]. On the one hand cortisone reduces the expression of vascular epithelial growth factor (VEGF), which inhibits abnormal angiogenesis. On the other hand it induces the secretion of the inhibitor of plasminogen, so that the cycle of re-bleeding and lysis is decelerated [3].

Several retrospective [21,27,30-36] and two prospective [25,28] studies about the use of cortisone in cSDH have been published. Two representative trials are discussed. In the first of the retrospective studies [30] 122 patients were enrolled and divided into two groups: Subjects with a good neurological condition (Markwalder grading score (MGS) 0 to 2) were assigned to the dexamethasone protocol, whereas patients with MGS 3 to 4 were assigned to a surgical protocol. Of the 122 patients, 101 were on dexamethasone, and 22 of them ultimately required a surgical drain (21.8%{). A favorable outcome (MGS 0, 1, or 2) was obtained in 96% and 93.9% of those treated with dexamethasone and surgical drain, respectively. The overall mortality rate was 0.8% and readmissions related to the hematoma reached 14.7%. The study medication comprised administration of 4 mg dexamethasone every 8 hours for 3 days, tapering to 1 mg every 3 days. In total the medication lasted for 36 days.

The second study [21] was of 142 patients treated with methylprednisolon and surgery, and 56 patient who only received surgery. The cortisone therapy decreased the risk of death threefold. Methylprednisolone was applied in a dosage of 0.5 mg/kg/day postoperatively for 30 days and was slowly tapered down subsequently.

The disadvantage of both cortisone protocols is their long duration and necessity of tapering the drug dose. There were medical complications in 27.8% of the dexamethasone group. Thus, the DRESH study has a drug application for 6 days only. The principal purpose is the proof of a practicable concomitant medication that has low incidence of side effects and is efficient, however.

Sun [25] prospectively enrolled a cohort of 112 patients and allocated them to four groups: burr-hole irrigation alone, dexamethasone with surgery, dexamethasone without surgery, and a small group treated only conservatively. Dexamethasone as a single treatment was administered four times daily (4 mg each) over 21 days, whereas in the dexamethasone plus surgery group the cortisone application started 48 hours preoperatively and was tapered over the next two weeks. Cortisone as a single treatment was equal to a combined treatment, because (re)operation was 4% in both groups. However, sole surgery resulted in a reoperation rate of 15%. Due to the small series no significant advantage of cortisone could be proven. There is a sense that a combined treatment is able to reduce the steroid dosage without affecting the recurrence rate.

Park [28] performed a fibrinogen and D-dimer analysis in cSDH and investigated 31 patients prospectively. All patients underwent burr-hole surgery and only one patient (3.2%) needed to undergo repeat surgery. This very low relapse rate is perhaps due to a concomitant dexamethasone medication for one week after the procedure.

The DRESH also uses the cortisone application postoperatively. Compared to a preoperative regime the advantage of a postoperative study drug administration is to additionally include patients who require urgent surgery. In spite of different study designs and surgical techniques the published data seem encouraging for a benefit of a steroid co-medication, even over a short period of time.

### Methodology

The DRESH study has a straightforward design: on the one hand an efficient concomitant treatment which should be evaluated to the highest evidence possible, and on the other hand the study dependent measures are perfectly integrated into the clinical routine. In the light of this aspect three points are worth mentioning: a clear study design and statistics, waived SAEs and a maximal time frame for randomization. Even patients who undergo emergency surgery can be randomized postoperatively. Most of the exclusion criteria are due to the use of dexamethasone or anticoagulant drugs. There is a simple primary endpoint defined without any surrogate parameter: reoperation. The reoperation rate should be decreased by 50%, because only 82 patients out of 820 (assumed reoperation rate of 10%) are affected and might benefit from the cortisone regime. In the case of a reduction in reoperation less than 50% more than 95% of the study population would take dexamethasone in vain. The surgical procedure has been standardized in order to achieve meaningful statistics.

The national authorities concerned have allowed waiving of particular SAEs, because dexamethasone has been used for decades. Hence, there is a clear understanding of the difference between drug-related and disease-related AEs. Evaluation of secondary objectives will be very lucrative because of the huge sample size. It will offer a refined knowledge of risk factors, the role of co-medication and radiologic findings. Nevertheless, the DRESH study has to clear two hurdles: to be the first RCT to prove significant efficacy for cortisone in the treatment of cSDH in general, and to test a reasonable medication dosage as a relapse prevention for surgical patients in particular.

In the event of significant results a powerful adjuvant therapy in preventing patients from reoperation is offered. For multi-morbid patients with high risk for surgery, a stand-alone therapy with dexamethasone is conceivable.

## Trial status

The anticipated start is at the beginning of February 2014.

## Abbreviations

ANOVA: Analysis of variance; CRF: Case report form; cSDH: Chronic subdural haematoma; CSF: Cerebrospinal fluid; CT: Computerized tomography; INR: International normalized ratio; IWRS: Interactive web response system; MGS: Markwalder grading score; MRI: Magnetic resonance imaging; RCT: Randomized controlled trial.

## Competing interests

The authors declare that they have no competing interests.

## Authors’ contributions

SE together with BR and RA created the study design. MC is responsible for the evaluation of the CT scans. WH calculated the statistics. LF, LS and YW adapted the protocol to Chinese conditions and elected the study cites. All authors read and approved the final manuscript.
